# Injury epidemiology in men’s professional team sports: is media analysis helpful?

**DOI:** 10.1007/s00402-020-03743-6

**Published:** 2021-01-11

**Authors:** Dominik Szymski, Leonard Achenbach, Volker Krutsch, Volker Alt, Rainer Meffert, Werner Krutsch, Kai Fehske

**Affiliations:** 1 Department of Trauma Surgery, University Medical Center Regensburg, Regensburg, Germany; 2 Department of Trauma-, Hand-, Plastic- and Reconstructive Surgery, Julius-Maximilians University Wuerzburg, Josef-Schneider-Strasse 2, 97080 Wuerzburg, Germany; 3 Department of Otorhinolaryngology, Paracelsus Medical University Nuremberg, Nuremberg, Germany; 4 SportDocsFranken, Nuremberg, Germany

**Keywords:** Professional, Injury, Media-based, Evidence, Validation, Severe injury

## Abstract

**Introduction:**

Epidemiological injury surveillance in professional sports is often based on online media analysis in order to collect necessary data. However, the validation of this study protocol is lacking. Therefore, this study aimed to identify the validity of injury surveillance in men’s professional team sports based on media reports.

**Methods:**

In a retrospective cohort study, the validity of media-data-relating injuries was investigated in participating teams of the highest two German divisions in men’s professional basketball (BB) and handball (HB) in the season 2018/2019. Injury protocols completed by the team physicians were compared to those of sports media injury reports.

**Results:**

The study population was composed of 133 athletes (54 BB and 79 HB). Of 343 injuries reported by the team physicians, 151 (44%) could be identified by means of sports media reports. Severe injuries (*n* = 75, 72%) were reported more likely in sports media compared to less severe injuries (*n* = 76, 32%, *p* < 0.00001). Odds ratio (OR) was 5.33 (95% CI 3.22–8.82). No differences regarding injury reporting could be seen between the two team sports.

**Conclusion:**

For severe injuries, media analysis may be a sufficient method for data collection in popular men’s professional ball sports. An underestimation of true injury prevalence lies within the range of previous reported investigations concerning the validation of injury surveillance methods. Non-severe injuries could not be verified via media analysis in professional handball and basketball.

## Introduction

Basketball and handball rejoice worldwide huge, annually growing, popularity and experience in recent years bigger interest in sport science [3, 25, 34]. Injuries are common in both sports and are well published in the literature [5, 7, 10, 33]. Besides other, both sports are predominantly accountable for a major amount of team sport injuries and showed high risk in all classes of performance [17, 22]. In comparison with other professional Olympic team sports, handball and basketball demonstrate both high injury rates [12, 19].

The physiology and biomechanics of movement in both sports are comparable and defined by cutting movements, pivot shifting, jumps and landing, as well as hard body confrontations and frequent intensity changes. In particular, while throwing body contact often takes place and increases injury risk, even though mechanics differ in this part of the game due to various types of scoring. However, the multidirectional composition and high intensity with body contact lead to huge load on musculoskeletal system of basketball and handball athletes [23, 32, 34, 35]. These analogical profiles of movement explain the similarities in injuries distribution. The injury pattern is predominated by injuries of the lower extremities, in particular of the knee and ankle [22, 28, 29].

Prospective cohort study is described as the recommended study design for epidemiological research in sports medicine [4, 15, 16]. Gathering of injury information often emphasizes complex due to low respond rates, in particular among professional sports athletes. In these situations, epidemiological injury surveillance in sports is often based on online media analysis in order to collect necessary data in professional sports. Free and available data by newspaper or other online media may be used for research in different types of sport and different issues, like return-to-play investigations, injury incidence, time-loss or sudden death among athletes [2, 9, 11, 31].

Nevertheless, adoption and usage of this protocol have to be taken carefully, correlated with medial attendance, and cannot be used for all types of professional sport. In 2020, the International Olympic Committee had published a Consensus Statement on Methods for Recording and Reporting of Epidemiological Data on Injury and Illness in Sports. The thereby described lack of study validation and comparability was found for 11 of 15 all-used protocols in sport injury surveillance. Only in four protocols validation studies were obtained [4].

Due to a lack of validation of this study protocol, this study aimed to identify the validity of injury surveillance based on media reports. Recently, first results of a media report analysis in professional football were published [21]. However, in other team sports data are missing. Thus, the aim of this study was to compare injury data based on media information to injury data reported by the team physicians. In addition, injury pattern according to localization and type in German men’s professional handball and basketball athletes were gathered and analysed with focus on commonalities and differences. This unique study design allows first time to make a statement with regard to power and quality of this type of methods. Based on previous results in professional men’s football [21], the hypothesis of this research was that severe injuries are commonly described in media concerning professional handball and basketball and could be a valid method for injury surveillance.

## Material and methods

This retrospective cohort study investigated the validity of injury diagnosis in media in the highest German men’s professional handball and basketball leagues in the season 2018/2019. All professional male basketball and handball teams in Germany attending to the two highest national leagues were included into this investigation.

Teams of the first or second national men’s leagues were contacted directly or by means of medical sports associations and invited to participate at the beginning of the season. For participation, team physicians were asked to submit all registered injuries in their respective teams to the study group at end of the season.

Only complete data sets of the participating teams were accepted for inclusion in this study. Incomplete data sets were excluded, as well as athletes younger than 18 years.

German online media portals in handball (https://www.handball-world.news/) and basketball (https://www.basketball.de/ and https://www.schoenen-dunk.de/), as well as social media (e.g. Facebook and Twitter) accounts of the participating teams, were analysed.

Injury classification and data analysis were done according to Krutsch et al. [22]. Ruptures (muscle ruptures and ligament tears), fractures, head concussions and dislocations were thereby graduated as severe, and distorsions, sprains, strains and skin lesions were assigned to not severe injuries.

Detailed information was given to every team, and informed consent was obtained from every study participant. The Ethics Committee of the University of Regensburg has approved the study (ID 17–895-101).

### Statistical analysis

Continuous data are expressed as mean and standard deviation (SD) and categorical data as frequency counts (percentages). Odds ratios accompanied by the corresponding 95% confidence interval (CI) are reported as effect estimates. Correlation between categorical variables was analysed with the Chi-square test and among continuous variables with the Student’s * t* test. The significance level was set to *p* < 0.05. All analyses were performed with IBM SPSS Statistics, version 24.0.

## Results

A total of 133 male athletes, of which 54 (41%) played basketball and 79 (59%) handball, were included. This comprised 5 teams of the first national league in BB and 4 teams of the first national league and 1 of the second national league in HB in Germany.

### Anthropometric data

The anthropometric characterization showed similar pattern in distribution of age and weight. BB players were taller compared to HB athletes (*p* = 0.0003) (Table 1).

**Table 1:** Anthropometric characteristics of the study population

	Basketball (*n* = 54)	Handball (*n* = 79)
Age (years)	26.8; SD 5.7	26.9; SD 4.8
Weight (kg)	93.2; SD 12.4	94.1; SD 9.1
Height (cm)	197.1; SD 9.5*	191.8; SD 7.1
BMI (kg/m^2^)	23.6; SD 2.3	24.5; SD 1.8

*p = 0.0003

### Injury pattern and media analysis

**Fig. 1:**
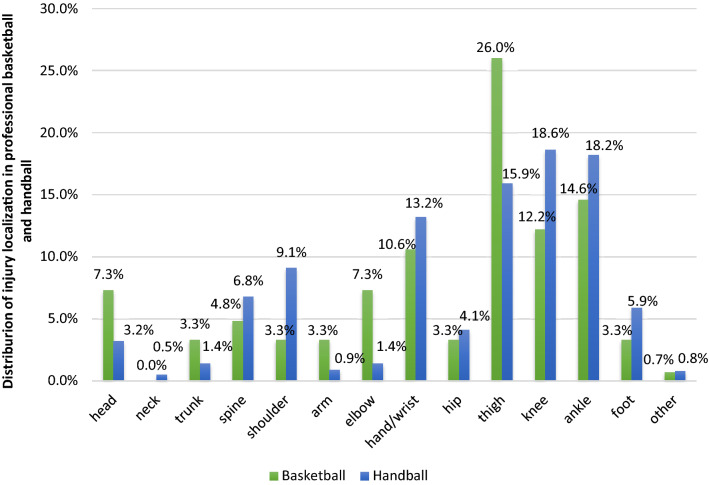
Distribution of injury localization in men’s professional basketball and handball athletes

**Fig. 2:**
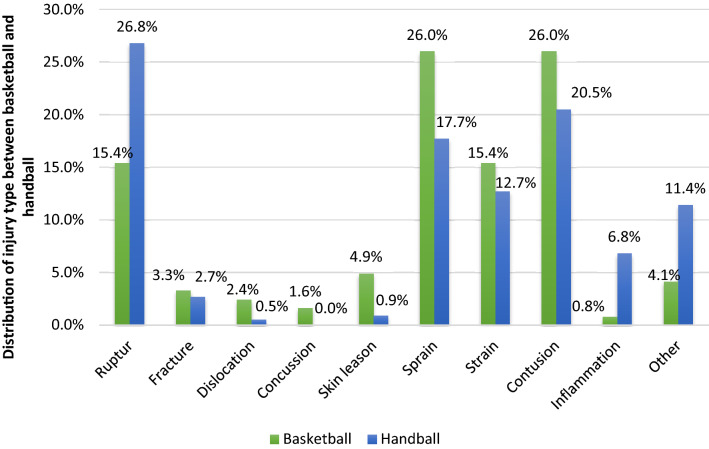
Distribution of injury type among professional handball and basketball athletes

In total, 343 injuries were registered by the team physicians in the season 2018/2019. Of these, 123 (36%) were sustained by basketball athletes and 220 (64%) by handball athletes. Of all injuries, predominantly non-severe injuries were reported (*n* = 238, 69%) in both sports. Basketball (*n* = 93, 76%) demonstrated a slightly higher number of non-severe injuries compared to handball (*n* = 145, 66%). The prevalence for severe injuries was almost doubled in handball (0.95 injuries in HB) compared to basketball with 0.56 injuries. Non-severe injuries showed less variety (HB: 1.72; BB: 1.84). Both types of sports showed similar pattern of injury localization with mainly the lower extremities affected. Thigh injuries, knee injuries and ankle injuries were predominantly reported (Fig. 1). Differences could be shown in distribution of injury types. While in handball predominantly ruptures (*n* = 59, 27%) and contusions (*n* = 45, 21%) occurred, in basketball mostly sprains and contusions (each *n* = 32, 26%) appeared (Fig. 2; Table 2).

**Table 2:** Injuries registered by team physicians

	Basketball (*n* = 54)	Handball (*n* = 79)	Total (*n* = 133)
Total injuries	123	220	343
	*n* (%)	*n* (%)	*n* (%)
Severe	30 (24)	75 (34)	105 (31)
Not severe	93 (76)	145 (66)	238 (69)

In both types of sport, almost half of injuries (*n* = 151, 44%) were registered in sports media. Both team sports demonstrated a very similar media coverage with basketball (*n* = 55; 45%) and handball (*n* = 96; 44%). Severe injuries (71%) were more often mentioned in media compared to not severe lesions (32%, *p* < 0.00001). Odds ratio (OR) for the total population was 5.33 (95% CI 3.22–8.82). Between handball and basketball, no significant differences were registered regarding the documentation of injury severity (Table 3). Media coverage of medical incidents differed vastly between the different teams: while the lowest rate lied at 30%, the highest rate of overall injuries reported in media was set at 77% (Table 4).

**Table 3:** Confirmed diagnosis of injuries by media analysis

	Basketball (*n* = 54)	Handball (*n* = 79)	Total (*n* = 133)
Diagnosis in media	*n* (%)	*n* (%)	*n* (%)
Total	55 (44.7)	96 (43.6)	151 (44.0)
Severe injuries	24 (80.0)**	51 (68.0)**	75 (71.4)**
Not severe injuries	31 (33.3)	45 (31.0)	76 (31.9)

**p < 0.00001

**Table 4:** Distribution of valid media diagnosis among the different included teams

Club	Diagnosis in media/total injuries (%)
1	11/18 (61)
2	5/9 (56)
3	10/24 (42)
4	22/57 (39)
5	7/15 (47)
6	28/81 (35)
7	21/36 (58)
8	7/16 (44)
9	17/57 (30)
10	23/30 (77)
Total	151/343 (44)

## Discussion

The most important finding of this study is that severe injuries in professional men’s team sports demonstrated a higher validity in media reports compared to less severe injuries. Official injury records of team physicians were compared with media data in online newspapers, sport journals and official club social media accounts. In particular, injuries, which were classified as severe [22], were 5.3 times better documented with a media coverage of 71%. In contrast, non-severe injuries could only be confirmed in 32% of all reported injuries. Both professional men’s team sports showed a similar media coverage. This medial interest could be explained by a higher impact on team performance of severe injuries and long-term consequences on starting formations and potential options during a game due to longer time loss. In particular, athletes in the starting formation and professionals are covered superior.

Like already used in previous studies, especially for severe injuries with long time loss, this method presents itself as a valid method for detection and data acquisition in professional sports. In the available literature, this principle was mainly used in medial highly represented sports with cultural/geographical differences, like football in Europe [24] or American football and ice hockey in the USA [8, 9, 14, 31]. Especially here due to the high attendance and interest of media, even better results of medial diagnosis could be shown.

A different method of data surveillance, but with equal aim, was used in professional men’s football by continuous collection of media data and validation of injury reports at the end of the season through the team physicians. Here overall 42.1% of all injuries could be confirmed. Severe injuries showed an equal pattern and were well documented in media with rates between 73.3% for fractures and 100% for joint dislocations. Hence, this research found in football similar results in analysis of media [21]. Our study extends these previous findings to the popular team ball sports handball and basketball.

Using media reports for research in professional sports has shown similar reporting rates compared to retrospective questionnaires of athletes and medical staff. An investigation among football players found a high rate of accuracy in retrospective reports of medical staff compared to player questionnaire with equal underestimation of incidence data between 19 (medical staff) and 24% (athletes) [6]. Our results had equal pattern of missed diagnoses, with 28.6% of underestimated severe injuries, to this retrospective monthly (medical staff) and annually (athletes) investigation. Also in skiing retrospective interviews with athletes at the end of the season revealed similar findings with 91% captured time-loss injuries. In this research, a bigger lack (61%) of accuracy was detected in registration by the medical team [13]. Reliability of results always underlies the setting of investigation and study personal. The implementation of health professionals during data collection showed greater rates and better coverage, in particular of light injuries (up to 8.8 times greater incidence), which were harder to identify for study assistance without medical background [36]. Junge et al. also observed the difference of a retrospective fulfilled injury questionnaire at the end of season by the athlete and weekly registration of injuries by a physician from their team. While among severe injuries no distinguish was determined, in total incidence and incidence of light injuries only one third of the appeared ones was indicated by athletes [18].

Overall, the method of data collection by media analysis for epidemiological injury surveillance in professional sports with high medial appearance turn outs to be a sufficient procedure. Equal to other validation investigations of retrospective data collection with monthly and annually interviews and injury forms, we found an underestimation of incidence for severe injuries between 20 and 32% [6, 13].

As second part of this investigation, also the gathered injury records were analysed and compared relating to localization and type between professional handball and basketball in Germany. Already in the literature the predominantly occurrence of lower extremity injuries in team sports was well described [20, 26]. Knee and ankle were demonstrated as primary localization of injuries in basketball and handball [3, 17, 22, 28, 30, 37]. We also found the majority of injuries located in the lower extremities, with focus on thigh (26.0%) and ankle (14.6%) among basketball and knee (18.6%) and ankle (18.2%) in handball. The high rate of thigh injuries in our study could be assumed by the high rate of contusions (26%), which also differs from the literature. But next to contusions, we also found accumulation of sprains (26%), equal to previous investigations [27, 37]. Furthermore, in handball the high rate of observed ruptures (26.8%) differs to other research. Majority of injury types was classified earlier as contusion and sprain and showed in addition similar pattern to our results [1, 22, 28].

Strengths of this research are the study design as prospective analysis of professional team sports with strong media coverage. Caused by the insurance system in Germany and accountant coding, the physicians' medical reports showed a high validity.

Low number of attending teams, a big cluster effect and the mixture of two different types of sport are limitations of this investigation. Another limitation is revealed in Table 4 by comparing the distribution of media analysis among the single teams. Depending on individual media policy of the clubs, a huge range with significant differences between teams was noticed. While some teams provided almost all injuries to media, others only supplied up to one third.

## Conclusion

Media analysis in order of injury surveillance in epidemiological investigations was shown to be a valid method for severe injuries in men’s professional handball and basketball, with a high media coverage of injuries of up to 71.4%. Though the injury underestimation of 28.6%, equal rates have been found in the literature concerning the validation of injury surveillance protocols. Non-severe injuries are not sufficiently documented in media and should not be used for epidemiological research.
